# PM_2.5_-induced oxidative stress increases intercellular adhesion molecule-1 expression in lung epithelial cells through the IL-6/AKT/STAT3/NF-κB-dependent pathway

**DOI:** 10.1186/s12989-018-0240-x

**Published:** 2018-01-12

**Authors:** Chen-Wei Liu, Tzu-Lin Lee, Yu-Chen Chen, Chan-Jung Liang, Shu-Huei Wang, June-Horng Lue, Jaw-Shiun Tsai, Shih-Wei Lee, Shun-Hua Chen, Yi-Fan Yang, Tzu-Yi Chuang, Yuh-Lien Chen

**Affiliations:** 1 Department of Anatomy and Cell Biology, College of Medicine, National Taiwan University, No. 1, Sec 1, Ren-Ai Road, Taipei, Taiwan; 2 Lipid Science and Aging Research Center, Kaohsiung Medical University, Kaohsiung, Taiwan; 3 Center for Lipid Biosciences, Kaohsiung Medical University Hospital, Kaohsiung, Taiwan; 4 Department of Family Medicine, College of Medicine and Hospital, Taipei, Taiwan; 5 Center for Complementary and Integrated Medicine, National Taiwan University Hospital, Taipei, Taiwan; 6 Department of Internal Medicine, Taoyuan General Hospital, Department of Health and Welfare, No.1492, Zhongshan Road, Taoyuan, Taiwan; 7 Department of Microbiology and Immunology, National Cheng Kung University, Tainan, Taiwan; 8 Department of Internal Medicine, National Taiwan University Hospital, College of Medicine, National Taiwan University, Taipei, Taiwan

**Keywords:** Particulate matters (PMs), Intercellular adhesion molecule-1 (ICAM-1), Reactive oxygen species (ROS), Interleukin-6 (IL-6), Inflammation

## Abstract

**Background:**

Epidemiological studies have shown that ambient air pollution is closely associated with increased respiratory inflammation and decreased lung function. Particulate matters (PMs) are major components of air pollution that damages lung cells. However, the mechanisms remain to be elucidated. This study examines the effects of PMs on intercellular adhesion molecule-1 (ICAM-1) expression and the related mechanisms in vitro and in vivo.

**Result:**

The cytotoxicity, reactive oxygen species (ROS) generation, and monocyte adherence to A549 cells were more severely affected by treatment with O-PMs (organic solvent-extractable fraction of SRM1649b) than with W-PMs (water-soluble fraction of SRM1649b). We observed a significant increase in ICAM-1 expression by O-PMs, but not W-PMs. O-PMs also induced the phosphorylation of AKT, p65, and STAT3. Pretreating A549 cells with N-acetyl cysteine (NAC), an antioxidant, attenuated O-PMs-induced ROS generation, the phosphorylation of the mentioned kinases, and the expression of ICAM-1. Furthermore, an AKT inhibitor (LY294002), NF-κB inhibitor (BAY11–7082), and STAT3 inhibitor (Stattic) significantly down-regulated O-PMs-induced ICAM-1 expression as well as the adhesion of U937 cells to epithelial cells. Interleukin-6 (IL-6) was the most significantly changed cytokine in O-PMs-treated A549 cells according to the analysis of the cytokine antibody array. The IL-6 receptor inhibitor tocilizumab (TCZ) and small interfering RNA for IL-6 significantly reduced ICAM-1 secretion and expression as well as the reduction of the AKT, p65, and STAT3 phosphorylation in O-PMs-treated A549 cells. In addition, the intratracheal instillation of PMs significantly increased the levels of the ICAM-1 and IL-6 in lung tissues and plasma in WT mice, but not in IL-6 knockout mice. Pre-administration of NAC attenuated those PMs-induced adverse effects in WT mice. Furthermore, patients with chronic obstructive pulmonary disease (COPD) had higher plasma levels of ICAM-1 and IL-6 compared to healthy subjects.

**Conclusion:**

These results suggest that PMs increase ICAM-1 expression in pulmonary epithelial cells in vitro and in vivo through the IL-6/AKT/STAT3/NF-κB signaling pathway.

**Electronic supplementary material:**

The online version of this article (10.1186/s12989-018-0240-x) contains supplementary material, which is available to authorized users.

## Background

Many developed and developing countries are experiencing severe and persistent haze pollution [1, 2]. The remarkable deterioration of air quality is mainly caused by rapid industrialization, urbanization, and population growth [3, 4]. Epidemiological studies have reported that smog episodes are significantly associated with higher total mortality and mortality from respiratory diseases [5, 6]. Particularly, fine particulate matters with size less than or equal to 2.5 mu (PM_2.5_) are the major risk factor for global burden of disorders [7]. Anthropogenic PM_2.5_ emitted from fossil fuel combustion contains harmful substances, such as organic chemicals that include polycyclic aromatic hydrocarbons (PAHs). PM_2.5_ is easily inhaled into the respiratory tract and accumulated in lung alveoli, where the toxic particles may result in both structural damage and lung function impairment. A previous study has shown that short-term exposure to high ambient air pollution increases airway inflammation and daily respiratory symptoms in patients with chronic obstructive pulmonary disease (COPD) [8]. However, the current understanding of the pathogenesis of PM_2.5_ is limited. Better understanding is needed to develop strategies for preventing or ameliorating lung injury to improve patient survival.

Epithelial cells initiate early inflammatory responses in the injured lung because of direct contact with PM_2.5_, which plays a central role in the pathogenesis of inflammatory and lung diseases [9]. One of the earliest pathological changes in inflammation and respiratory diseases is the expression of cell adhesion molecules such as intercellular adhesion molecule-1 (ICAM-1) on epithelial cells [10]. The expression of ICAM-1 on epithelial cells mediates monocyte/macrophage adherence and infiltration into inflammatory sites and pulmonary lesions [11]. Elevated plasma or serum concentrations of soluble ICAM-1 (sICAM-1) have been reported in patients with pulmonary diseases [12]. A complex array of intracellular signaling pathways involving reactive oxygen species (ROS), mitogen-activated protein kinases (MAPKs), and transcription factors may be required in the upregulation of ICAM-1 expression [13–15]. Moreover, several studies indicate that the upregulation of ICAM-1 expression on epithelial cells is closely associated with pro-inflammatory cytokines, such as interleukin-6 (IL-6), interleukin-1β (IL-1β), and tumor necrosis factor-α (TNF-α) [9, 16, 17].

Little is known about the effects of PMs on adhesion molecule expression under inflammatory conditions and the mechanisms of these effects. Better understanding in this respect might provide important insights into the therapy of COPD patients. In the present study, biomarkers related to inflammation and oxidative stress were experimentally investigated in vitro and in vivo to clarify PMs-induced lung inflammation and the related mechanisms. First, an in vitro study was performed to investigate the expression of ICAM-1 and the related signals (MAPKs/AKT/STAT3/NF-κB) in PMs-stimulated A549 cells. Second, an in vivo study was carried out to investigate the expression of ICAM-1 and IL-6 in PMs-treated wild-type (WT) and IL-6 KO mice. Lastly, a human study was done to investigate the plasma levels of sICAM-1 and IL-6 in COPD patients.

## Methods

### Preparation of water-soluble PMs (W-PMs) and organic-extractable PMs (O-PMs)

The PMs were standard reference material 1649b (SRM1649b) and were purchased from NIST (MD, USA). The PMs were composed of urban dust. The characteristics of the PMs have been described previously [18]. A total of 50 mg of PMs were dispersed in 1 ml of milli-Q water or 1 ml of dimethyl sulfoxide (DMSO), shaken, and ultra-sonicated for 2 h in an ultrasonic bath. The samples were centrifuged at 13000×g for 30 min to collect the supernatant as water-soluble PMs (W-PMs) and organic-extractable PMs (O-PMs). All extracts were stored at −20 °C until biological analysis.

### Cell culture

A549 cells (neoplastic transformation of human lung type II epithelial alveolar cells) and U937 cells (human monocytic leukemia cells) were purchased from American Type Culture Collection (VA, USA). A549 cells were cultured in Dulbecco’s Modified Eagle Medium (DMEM) (Life Technology, NY, USA) containing 10% fetal bovine serum (FBS, Life Technology) and 1% penicillin/streptomycin. U937 cells were cultured in RPMI 1640 medium (Life Technology) supplemented with 10% FBS and 1% penicillin/streptomycin. The cells were grown in a humidified atmosphere comprising 95% air and 5% CO_2_ at 37 °C.

### Cytotoxicity assay

The MTT [3-(4, 5-dimethylthiazol-2-yl)-2, 5-diphenyltetrazolium bromide] assay was used to evaluate the effects of O-PMs and W-PMs on the cell viability of A549 cells. The cells were seeded in 96-well plates at a density of 1 × 10^4^ cells per well in 100 μl of medium and cultured for 24 h, and then the cells were treated with 0, 25, 50, 100, 200, and 400 μg/ml of PMs for 24, 48, and 72 h. After the exposure, 20 μl of MTT (1 mg/ml in PBS) was added to each well and incubated continuously for 2 h at 37 °C. The cells were then treated with 100 μl of DMSO. The absorbance was measured at 570 nm using a microplate spectrophotometer (Thermo MK3, MA, USA). The data are expressed as a percentage with respect to the value obtained for the solvent control (0.1% DMSO), which was set to 100%. Experiments were performed in triplicate and repeated 3 times.

### Measurement of ROS production

A 2′, 7′-dicholorofluorescein-diacetate (DCFH-DA) probe was used to determine the level of intracellular ROS production by PMs-treated A549 cells on 12-well plates. Confluent A549 cells were treated with 50 or 100 μg/ml of W-PMs or O-PMs for 24 h, followed by the addition of 10 μM DCFH-DA for 20 min at 37 °C in the dark. The cells were then washed twice with ice-cold PBS. The fluorescence was observed with a fluorescence microscope (excitation, 488 nm; emission, 519 nm) and analyzed with a FACScan flow cytometer (Becton Dickinson, CA, USA).

### Flow cytometry for the detection of ICAM-1 on the epithelial cells

The surface expression of ICAM-1 on A549 cells was evaluated by flow cytometry as reported previously [19]. Briefly, the cells were treated or untreated for 1 h with 10 μM of Bay11–7082 (NF-κB inhibitor), LY294002 (AKT inhibitor), Stattic (STAT3 inhibitor), SB203580 (p38 inhibitor), or SP600125 (JNK inhibitor). They were then treated with 100 μg/ml of O-PMs for 24 h and washed twice with PBS. After trypsinization, suspended A549 cells (10^5^ cells) were incubated in 100 μl of PBS containing 5 μl of FITC-conjugated anti-human ICAM-1 antibody (Dako, CA, USA) or FITC-conjugated control IgG for 15 min. The cells were then suspended in 500 μl of PBS and analyzed using a FACScan flow cytometer. The experiments were performed in triplicate and repeated 3 times.

### Epithelial cell-leukocyte adhesion assay

A549 cells grown in 24-well plate were untreated or pretreated for 1 h with 10 μM of Bay11–7082 (NF-κB inhibitor), LY294002 (AKT inhibitor), Stattic (STAT3 inhibitor), 5 mM of N-acetyl cysteine (NAC), 1 μg/ml of anti-ICAM-1 antibody, or p65 siRNA. The cells were then treated with or without 100 μg/ml of O-PMs for 24 h. The U937 cells were labeled for 1 h at 37 °C with 10 mM BCECF/AM (Boehringer Mannheim, Mannheim, Germany) in DMSO and then suspended in the same medium used for culturing the A549 cells. For the test, 10^6^ labeled U937 cells were added to 10^6^ adherent PM-treated A549 cells in a 24-well plate and incubated for 1 h. The nonadherent cells were removed by two gentle washes with PBS, and the number of bound U937 cells were counted using fluorescence microscopy.

### Antibody array analysis

A human cytokine antibody array (RayBiotech, GA, USA) was used to identify cytokines secreted from A549 cells with or without O-PMs treatment. The experimental procedures were performed according to the manufacturer’s instructions. The signal intensity of each spot was analyzed using ImageJ software and normalized to the average of the positive control spots to determine the variation of the cytokines.

### Preparation of cell lysates and western blot analysis

Western blot analyses were performed as described previously [20]. Cells were harvested with lysis buffer (20 mM Tris-HCl, 150 mM NaCl, 1 mM EDTA, 1 mM EGTA, 1% Triton X-100, and 1 mM phenylmethylsulfonyl fluoride) supplemented with protease and phosphatase inhibitor (Thermos Fisher Scientific, IL, USA). The lysates were then centrifuged at 14000×g for 30 min at 4 °C. Cytoplasmic and nuclear fractions were extracted using cytoplasmic extraction and NE-PER Nuclear reagents (Thermo Fisher Scientific), respectively. Equal amounts of the supernatants (20 μg of protein) were subjected to sodium dodecyl sulfate (SDS)-polyacrylamide gel electrophoresis and transferred to polyvinylidene fluoride membranes (Pall Corporation, NY, USA). The membranes were then incubated for 30 min at room temperature (RT) with 5% nonfat milk in Tris-buffered saline containing 0.2% Tween 20 to block nonspecific binding of antibodies. All dilutions of antibodies used were in TBST.

The membranes were incubated overnight at 4 °C with rabbit antibodies against human ICAM-1, phospho-STAT3, t-STAT3, IL-6 (Abcam, Cambridge, UK; 1:5000 dilution), phospho-JNK, t-JNK (Cell Signaling Technology, MA, USA; 1:2000 dilution), phospho-ERK1/2, t-ERK1/2 (Cell Signaling Technology; 1:8000 dilution)*,* phospho-p38, t-p38 (Santa Cruz Biotechnology, TX, USA; 1:8000 dilution), t-p65, phospho-p65 (Epitomics, CA, USA; 1:1000 dilution), and Lamin A, α-Tubulin, β-actin (Epitomics; 1:5000 dilution). They were then incubated for 1 h at RT with horseradish peroxidase-conjugated goat anti-rabbit IgG antibodies (Sigma, MO, USA; 1:2000 dilution), which are bound antibodies that are detected using chemiluminescence reagent Plus (NEN, MA, USA). Images were visualized by a UVP BioSpectrum 600 imaging system (UVP, CA, USA), and the intensity of each band was quantified using a densitometer. The antibody against GAPDH (Santa Cruz Biotechnology; 1:3000 dilution) served as a loading control.

### siRNA transduction

The specific Accell SMART pool siRNAs (Dharmacon, Inc., PA, USA) were used to target p65 or IL-6 to silence p65 or IL-6, respectively. A 100 μM stock of siRNA was prepared in RNase-free water and stored at −20 °C. A549 cells were cultured in a 6-well plate at 70–80% confluence for 24 h. The culture medium in each well was then added with 1 μM of p65 or IL-6 siRNA in Turbofect™ (Thermo Fisher Scientific). After siRNA transfection for 24 h, cells were stimulated with 100 μg/ml of O-PMs for 24 h. The downregulation of p65 expression in cell lysates were confirmed by Western blot. The downregulation of IL-6 expression in conditioned medium (CM) was also confirmed by ELISA.

### Human participants study

Blood was obtained from 8 patients who had been diagnosed with COPD and 8 healthy subjects without a history of COPD at General Taoyuan Hospital, Taoyuan, Taiwan. All COPD patients had a history of smoking. None of the healthy subjects had ever been smokers. Written informed consent was obtained from each individual. The study protocol conformed to the ethical guidelines of the 1975 Declaration of Helsinki and was approved by the Ethics Committee of Taoyuan General Hospital (TYGH99025). Blood was collected in sterile test tubes with heparin and centrifuged at 1000×g for 10 min and stored at −80 °C until subsequent experiments.

### sICAM-1 and IL-6 in conditioned media and in plasma of mice and humans by enzyme-linked immunosorbent assay (ELISA)

Conditioned media were collected from A549 (2 × 10^5^) with and without 100 μg/ml of O-PMs for 24 h. The plasma was collected from mice and patients. The sICAM-1 expression was determined using ELISA kits (R&D Systems, MN, USA). The ELISA kits for IL-6 expression from humans or mice were purchased from BioLegend (CA, USA) and R&D Systems, respectively. The experimental procedures were performed according to the manufacturer’s protocols. Cell samples were run in triplicate and repeated three times. The plasma was collected from 8 patients with COPD and 8 healthy subjects. Plasma was also collected from mice (six mice/group) after 7 days and 14 days after PMs treatment. The absorbance was measured at 450 nm on an EL808 microplate absorbance reader (BioTek, VT, USA).

### Immunofluorescent staining

A sterilized coverslip with 0.1% gelatin coating was placed into a well of a 24-well plate. A549 cells were seeded onto the coverslip at a density of 1 × 10^5^ cells/ml. To examine ICAM-1 expression in situ, confluent A549 cells were treated with 100 μg/ml of W-PMs or O-PMs for 24 h. The media were then removed, washed with PBS, fixed with 4% formaldehyde for 15 min, and permeabilized with 0.3% Triton X-100 for 1 min at RT. The cells were blocked in PBS containing 1% bovine serum albumin (BSA) for 1 h at RT. The cells were incubated with ICAM-1 (1:500 dilution in PBS; Abcam) at 4 °C overnight. Unbounded antibody was washed with PBS and DyLight® 488 conjugated secondary antibodies (1:1000 dilution in PBS, Abcam) for 1 h at RT. The cells were counterstained with 1 μg/ml of DAPI for 5 min. The results were observed and photographed by fluorescence microscopy.

### Animal model of PMs intratracheal instillation

All procedures involving experimental animals were performed in accordance with the guidelines for animal care of National Taiwan University (ICCUC: 20,160,235) and complied with the Guide for the Care and Use of Laboratory Animals, NIH publication No.86–23, revised 1985. Male C57BL/6 wild type (WT) mice and IL-6 knockout (KO) mice were purchased from National Taiwan University (Taipei, Taiwan). The mice were 8–12 weeks old and weighed between 20 and 35 g. A method of intratracheal instillation of PMs was performed on the mice, which was modified from previous reports [21, 22]. Briefly, mice were anesthetized using inhaled 2% isoflurane. The necks of the mice were shaved, and the surgical area was sterilized with 75% alcohol. A vertical 5-mm incision was made, and the trachea was exposed. The anterior wall of the trachea between the second and third tracheal cartilage rings was punctured using an insulin syringe at a 45° angle to avoid damaging the posterior wall.

PMs was thawed at RT and diluted in sterile PBS to final concentrations of 200–350 μg/100 μl. A suspension containing 100 μl of PMs (200–350 μg/mouse) in sterile PBS was slowly instilled intratracheally, followed by 100 μl of clean air (*n* = 6 in each group at the determined time). In addition, 50 μl of NAC (3–5 mg/mouse, 150 mg/kg body weight) in PBS was injected intraperitoneally into WT mice 1 day before the administration of PMs. The dose of NAC used in the present study was followed with the previous report [23]. The control mice were instilled with an equal volume of PBS. The forelegs of the mice were held to keep the animal upright for 10 s, and then the mice were rotated to help the instilled solution enter the lungs. The skin incision was sutured after instillation. When normal behavior was restored, the mice were placed back into their cages. They were then anesthetized by intraperitoneal injection of Zoletil/Xylazine (2 mg/kg + 5 mg/kg) and sacrificed at Day 7 or Day 14 after intratracheal instillation. Blood was collected from each group and the plasma was used to measure the levels of sICAM-1 and IL-6 by ELISA.

A part of the lung tissues was immersion-fixed with 4% buffered paraformaldehyde and embedded in paraffin for immunohistochemistry. The remaining larger portion was immediately frozen in liquid nitrogen for protein isolation to detect the levels of ICAM-1 and IL-6 by Western blot. Briefly, lung tissue was lysed by lysis buffer (20 mM Tris-HCl, 150 mM NaCl, 1 mM EDTA, 1 mM EGTA, 1% Triton X-100, and 1 mM phenylmethylsulfonyl fluoride) supplemented with protease and phosphatase inhibitor. The lysates were then centrifuged at 14000×g for 30 min at 4 °C. Supernatants were stored at −80 °C. Blood was rapidly collected from each group in heparinized tubes and then centrifuged at 3000 rpm for 15 min, and the plasma was used to measure the levels of sICAM-1 and IL-6 by ELISA.

### Immunohistochemistry

To determine the levels of ICAM-1 and IL-6 expression in lung tissues, 5-μm-thick sections were incubated overnight with ICAM-1 or with IL-6 antibodies (1:100 dilutions, Abcam) at 4 °C. Subsequently, the sections were incubated with biotin-conjugated goat anti-rabbit IgG (1:200 dilutions, Vector lab, Cambridgeshire, UK) for 1 h at RT. The sections were then stained with 3, 3-diaminobenzidine tetrahydrochloride (DAB), counterstained with hematoxylin, and examined by light microscope.

### Transmission electron microscopy

A549 cells treated with 100 μg/ml O-PMs for 24 h were collected by centrifugation and washed with PBS, followed by fixing with 2% glutaraldehyde and 2% paraformaldehyde in PBS for 1 min, and post-fixing with 1% osmic acid for 30 min. The samples were then dehydrated in graded ethanol, washed with propylene oxide, and embedded in epoxy resin. Ultrathin sections cut in a Reichert ultramicrotome were stained with lead citrate and uranyl acetate and were examined in a HITACHI H-7100 at 100 kV.

### Statistical analysis

All data are expressed as the mean ± SEM. The data concerning cytotoxicity assay were analyzed using three-way ANOVA (PM dose, PM type and time-course as fixed factors) and animal study were analyzed using two-way ANOVA (mouse species and time-course as fixed factors) with the Tukey-Kramer HSD comparison test. All other experiments were determined by one-way-ANOVA and a Dunnett post hoc test. A value of *P* < 0.05 was considered statistically significant.

## Results

### The cytotoxicity, ROS generation and monocyte adherence to A549 cells were more severely affected by O-PMs than by W-PMs treatment

**Fig. 1:**
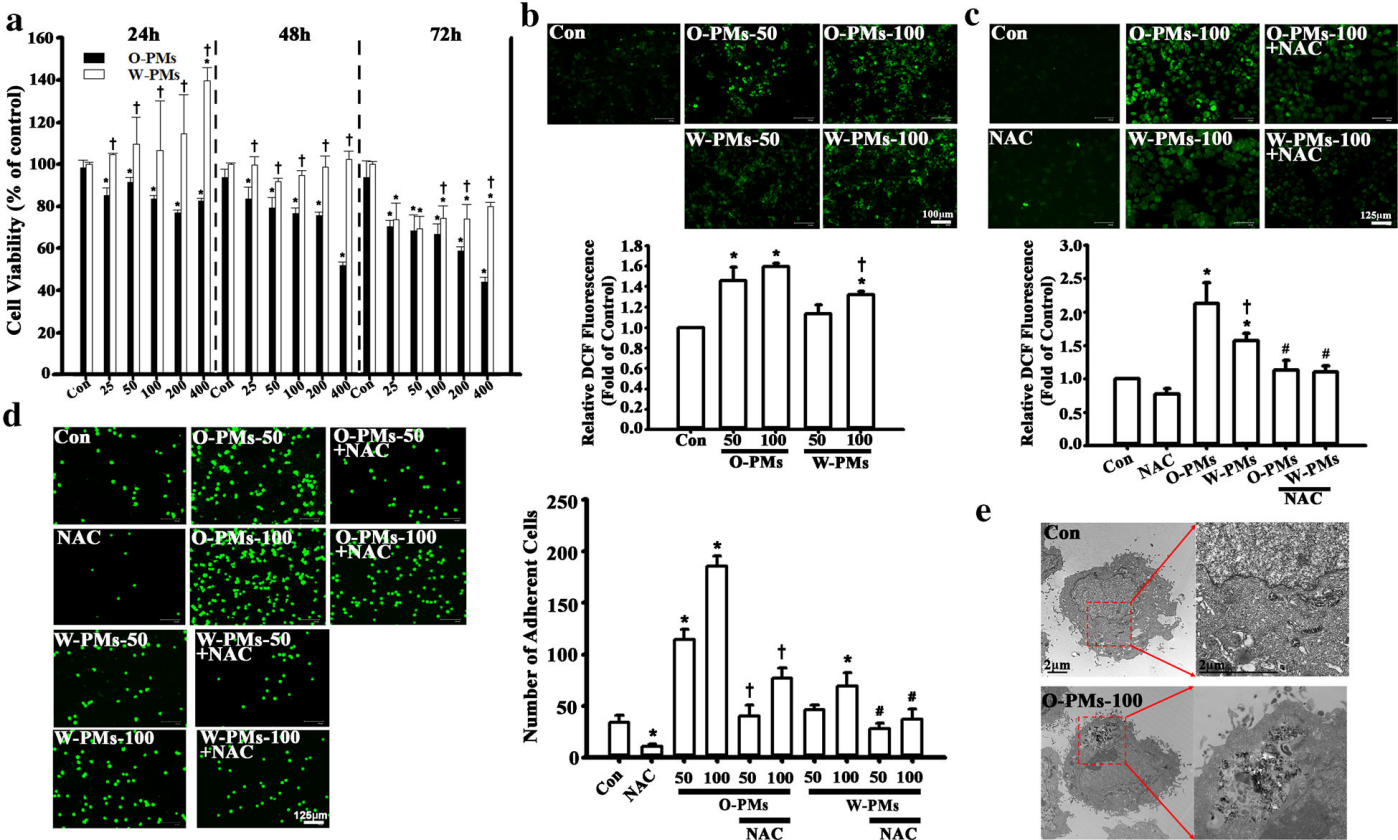
Cytotoxicity, ROS generation, and monocyte adherence to A549 cells were more severely affected by O-PM than by W-PM treatment. **a** The cytotoxic effects of O-PMs (organic solvent-extractable fraction of PMs) and W-PMs (water-soluble fraction of PMs) on A549 cells at the concentrations of 25, 50, 100, 200, and 400 μg/ml for 24, 48 and 72 h were examined by MTT assay. The data are expressed as a percentage of that obtained for DMSO-treated or media-treated cells, which was set to 100%. *p < 0.05 vs. the control cells in the corresponding groups. †*p* < 0.05 vs. O-PMs. **b** The intracellular ROS levels in A549 cells after the exposure of 50 or 100 μg/ml of O-PMs or W-PMs for 24 h were determined using DCFH-DA by fluorescent microscope and flow cytometer. Bar = 100 mu. **c** A549 cells were pretreated with 5 mM of N-acetyl cysteine (NAC) for 1 h and then treated with 100 μg/ml of O-PMs or with W-PMs for 24 h. The intracellular ROS levels were determined using DCFH-DA by fluorescent microscope and flow cytometer. Bar = 125 mu. **d** Representative fluorescence photomicrographs and quantitative data showing the effect of O-PMs and W-PMs-treated A549 cells on the adhesion of fluorescein-labeled U937 cells. A549 cells were treated with or without 5 mM NAC for 1 h, and were then treated with or without 50 or 100 μg/ml of O-PMs or W-PMs for 24 h. The number of bound U937 cells was counted by fluorescence microscopy. Values are the mean ± SD of three independent experiments. *p < 0.05 vs. con. †*p* < 0.05 vs. O-PMs-50 or O-PMs-100, respectively. #*p* < 0.05 vs. W-PMs-50 or W-PMs-100, respectively. Bar = 125 mu. **e** TEM images of A549 cells. O-PMs were located in the cytoplasm of A549 cells after 100 μg/ml O-PMs exposure for 24 h. Bar = 2 mu

MTT assay showed that O-PMs and W-PMs significantly decreased the viability of A549 cells treated with different concentrations of the O-PMs or W-PMs for 24, 48, and 72 h. However, a higher concentration and longer incubation time of W-PMs was required to induce cytotoxicity (Fig. 1a). The potential of PMs inducing intracellular ROS generation in A549 cells was evaluated according to the DCFH-DA intensity. Cells treated with 50 or 100 μg/ml of PMs for 24 h significantly increased ROS production according to fluorescence microscopy and flow cytometry (Fig. 1b). O-PMs-induced ROS production was higher than that in W-PMs. Pretreatment with NAC, an antioxidant, significantly decreased PMs-induced ROS production (Fig. 1c).

To explore the effects of PMs on the epithelial cell-leukocyte interaction, we examined the adhesion of U937 cells to PMs-treated cells. As shown in Fig. 1d, control confluent A549 cells (Con) incubated with U937 cells for 1 h showed minimal binding, but adhesion was significantly increased when the cells were pretreated with PMs for 24 h. Importantly, the number of monocytes that adhered to O-PMs-treated cells was 2-times higher than those treated with W-PMs. Furthermore, NAC pretreatment significantly decreased O-PMs-increased monocyte adhesion (Fig. 1d). The TEM images of O-PMs-treated A549 cells show aggregates of ultrafine particles, which were either surrounded by cell membrane or freely present in the cytoplasm (Fig. 1e).

### O-PMs increased ICAM-1 expression in A549 cells, but not W-PMs

**Fig. 2:**
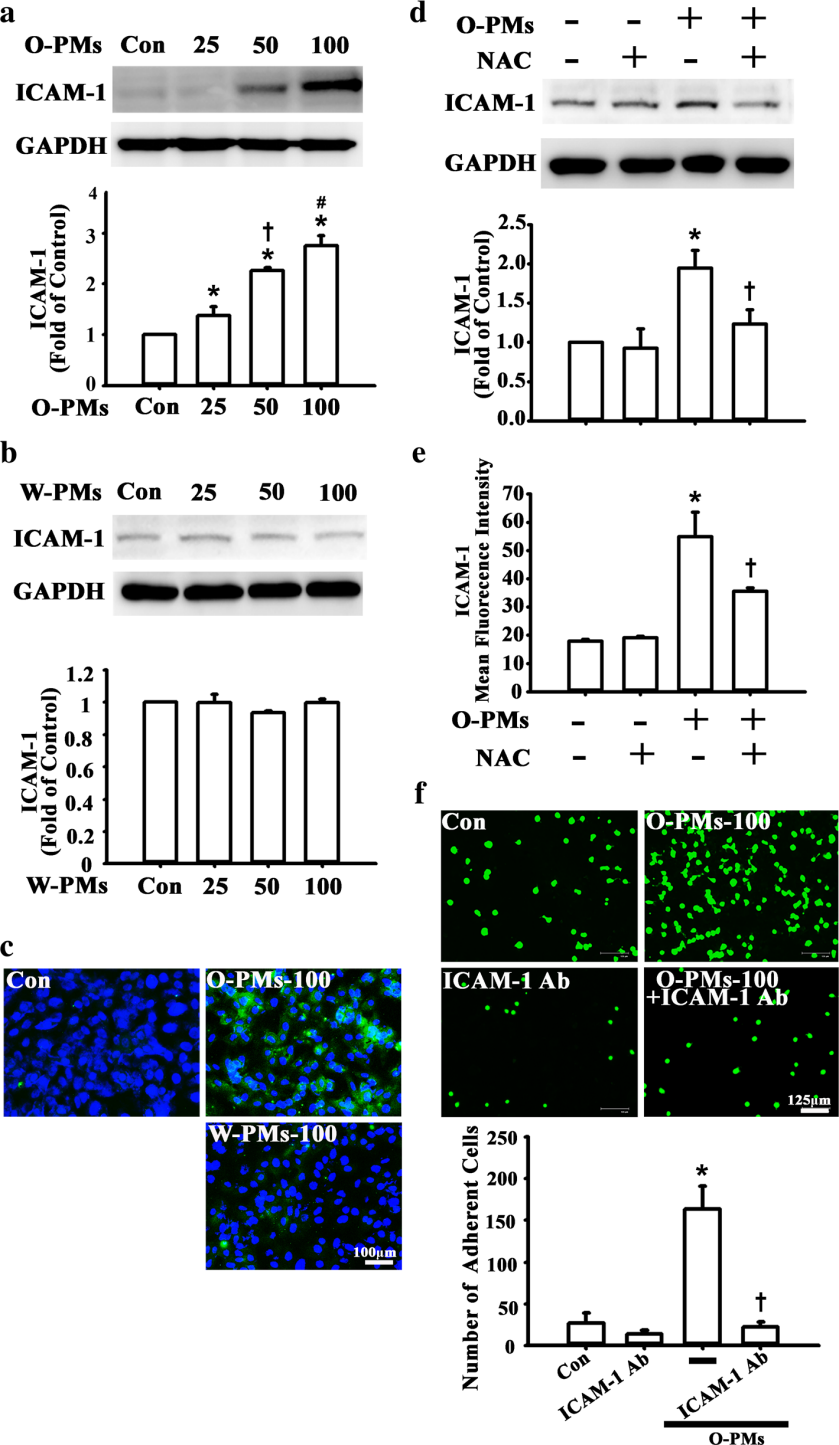
O-PMs-increased ICAM-1 Expression in A549 Cells, but not W-PMs. **a**-**b** A549 cells were exposed to 0, 25, 50 or 100 μg/ml O-PMs (**a**) or W-PMs (**b**) for 24 h. ICAM-1 protein in cell lysates was measured by Western blot as described in the Methods section. GAPDH was used as the loading control. *p < 0.05 vs. untreated cells. †*p* < 0.05 vs. O-PMs-25. #*p* < 0.05 vs. O-PMs-50. **c** The distribution of ICAM-1 was analyzed by immunofluorescent staining. Bar = 100 mu. **d**-**e** A549 cells were pretreated with 5 mM NAC for 1 h and then treated with 100 μg/ml O-PMs for 24 h. ICAM-1 protein in cell lysates was measured by Western blot (**d**) and the ICAM-1 surface expression was detected by flow cytometer (**e**). *p < 0.05 vs. Con. †*p* < 0.05 vs. O-PMs. **f** Representative fluorescence photomicrographs and quantitative data showing the effect of ICAM-1 expression on the adhesion of BCECF-AM-labeled U937 cells to O-PMs-treated A549 cells. A549 cells were cotreated with 100 μg/ml O-PMs and with 1 μg/ml anti-ICAM-1 antibody or anti-IgG for 24 h. Bar = 125 mu. *p < 0.05 vs. Con. †*p* < 0.05 vs. O-PMs

ICAM-1 is a type of adhesion molecule that is thought to mediate monocyte binding to epithelial cells during the development of pulmonary diseases [24]. To examine whether PMs enhanced ICAM-1 expression, A549 cells were incubated with 0–100 μg/ml of PMs for 24 h, and then ICAM-1 expression in cell lysates was measured by Western blot. As shown in Fig. 2a, O-PMs treatment significantly increased ICAM-1 expression in a dose-dependent manner (by 1.3 ± 0.2 times compared to untreated control levels at 25 μg/ml, 2.2 ± 0.1 at 50 μg/ml, 2.7 ± 0.2 at 100 μg/ml). In contrast, W-PMs did not have any effect on ICAM expression in the cell lysates (Fig. 2b). These results are consistent with the immunofluorescent images (Fig. 2c).

Next, ICAM-1 expression in the cell lysates and the surface expression of ICAM-1 in response to O-PMs were increased by Western blot and flow cytometry, respectively. NAC pretreatment attenuated the O-PMs-induced ICAM-1 expression (Fig. 2d and e). Furthermore, ICAM-1 antibody treatment significantly decreased the monocyte adhesion to O-PMs-treated epithelial cells (Fig. 2f).

### O-PMs induced ROS production and ICAM-1 expression in A549 cells through AKT/STAT3/p65 phosphorylation

**Fig. 3:**
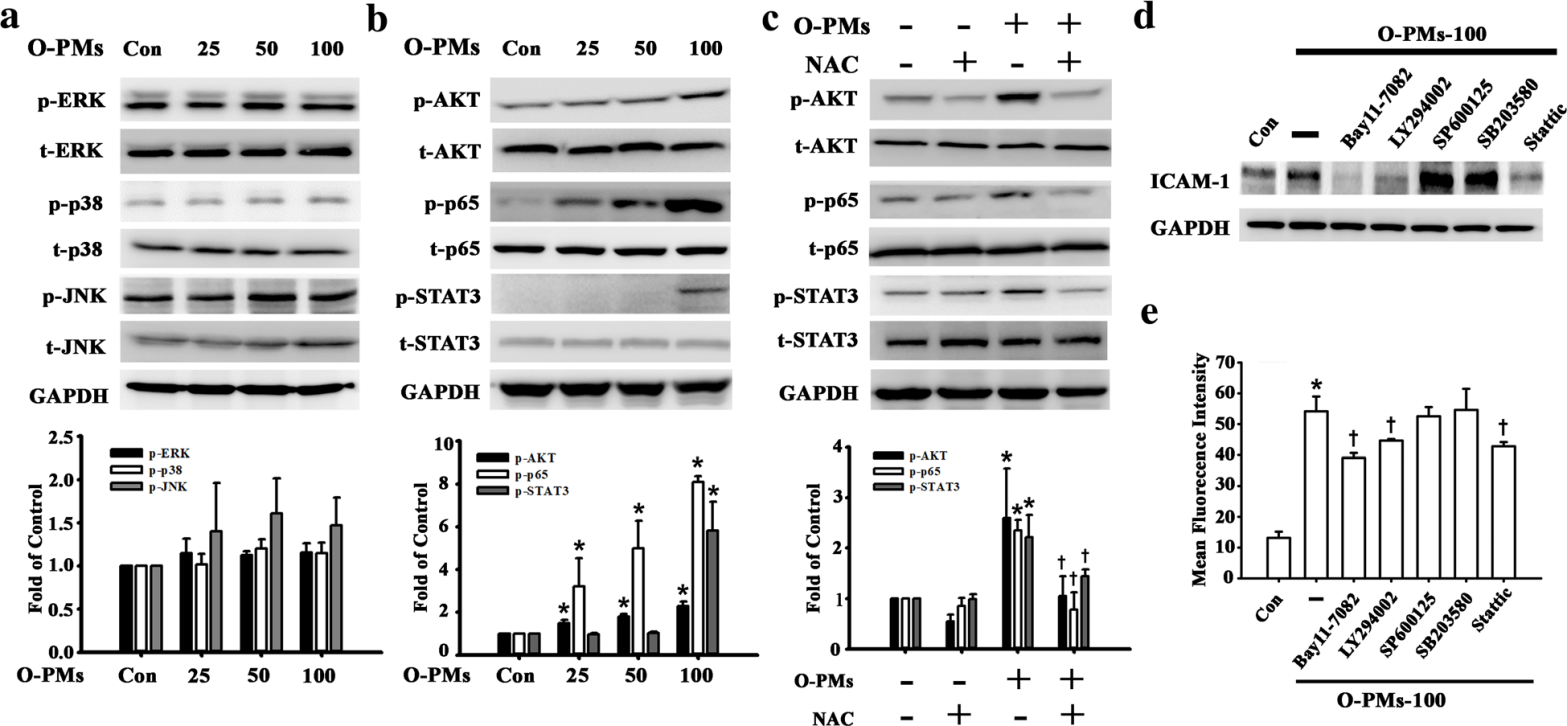
O-PMs induced ROS production and ICAM-1 expression in A549 cells through AKT/STAT3/p65 phosphorylation. **a**-**b** The effects of O-PMs treatment on the phosphorylation of (**a**) ERK, p38, JNK (**b**) AKT, p65, and STAT3 in A549 cells. A549 cells were exposed to 0, 25, 50 or 100 μg/ml O-PMs for 24 h. Cell lysates were analyzed by Western blot with antibodies against p-ERK, t-ERK, p-JNK, t-JNK, p-p38, t-p38, p-AKT, t-AKT, p-p65, t-p65, p-STAT3, and t-STAT3. **c** The effects of NAC treatment on the phosphorylation of AKT, p65 and STAT3 in O-PMs-treated A549 cells by Western blot. A549 cells were pretreated with 5 mM of NAC for 1 h and then treated with 100 μg/ml O-PMs for 24 h. *p < 0.05 vs. Con. **d**-**e** A549 cells were pretreated with BAY11–7082 (10 μM), LY294002 (10 μM), SP600125 (10 μM), SB203580 (10 μM), or Stattic (10 μM) for 1 h and then treated with 100 μg/ml O-PMs for 24 h. ICAM-1 protein in cell lysates was measured by Western blot (D) and the ICAM-1 surface expression was detected by flow cytometry (E). *p < 0.05 vs. Con. †*p* < 0.05 vs. O-PMs

The induction of ICAM-1 expression by inflammatory cytokines is mediated by several signaling pathways that involve MAPKs, AKT, STAT3, and p65 [25, 26]. First, we investigated whether the O-PMs-induced increase in ICAM-1 expression in A549 cells was mediated by activation of MAPKs. As shown in Fig. 3a, O-PMs treatment for 24 h did not induce phosphorylation of ERK, p38, and JNK. In contrast, the phosphorylation of AKT, STAT3, and p65 was significantly increased after exposure to 25–100 μg/ml of O-PMs in a concentration-dependent manner (Fig. 3b). The expression of p-p65 was more robust than p-AKT and p-STAT3. The phosphorylation of AKT, p65, and STAT3 was significantly attenuated after pretreatment with NAC (Fig. 3c).

These findings indicated that ROS production is involved in the activation of the AKT, NF-κB, and STAT3 pathways in response to O-PMs. Moreover, the increase in ICAM-1 expression in response to O-PMs treatment was inhibited by pretreatment with Bay11–7082 (NF-κB inhibitor), LY294002 (AKT inhibitor), and Stattic (STAT3 inhibitor), but not with SB203580 (p38 inhibitor) or SP600125 (JNK inhibitor) by Western blot and flow cytometry (Fig. 3d and e). These data suggest that O-PMs-induced ROS production is involved in the activation of the AKT, NF-κB, and STAT3 pathways and these pathways are capable of stimulating ICAM-1 expression in A549 cells.

### NF-κB p65 activation was involved in O-PMs-induced ICAM-1 expression in A549 cells

**Fig. 4:**
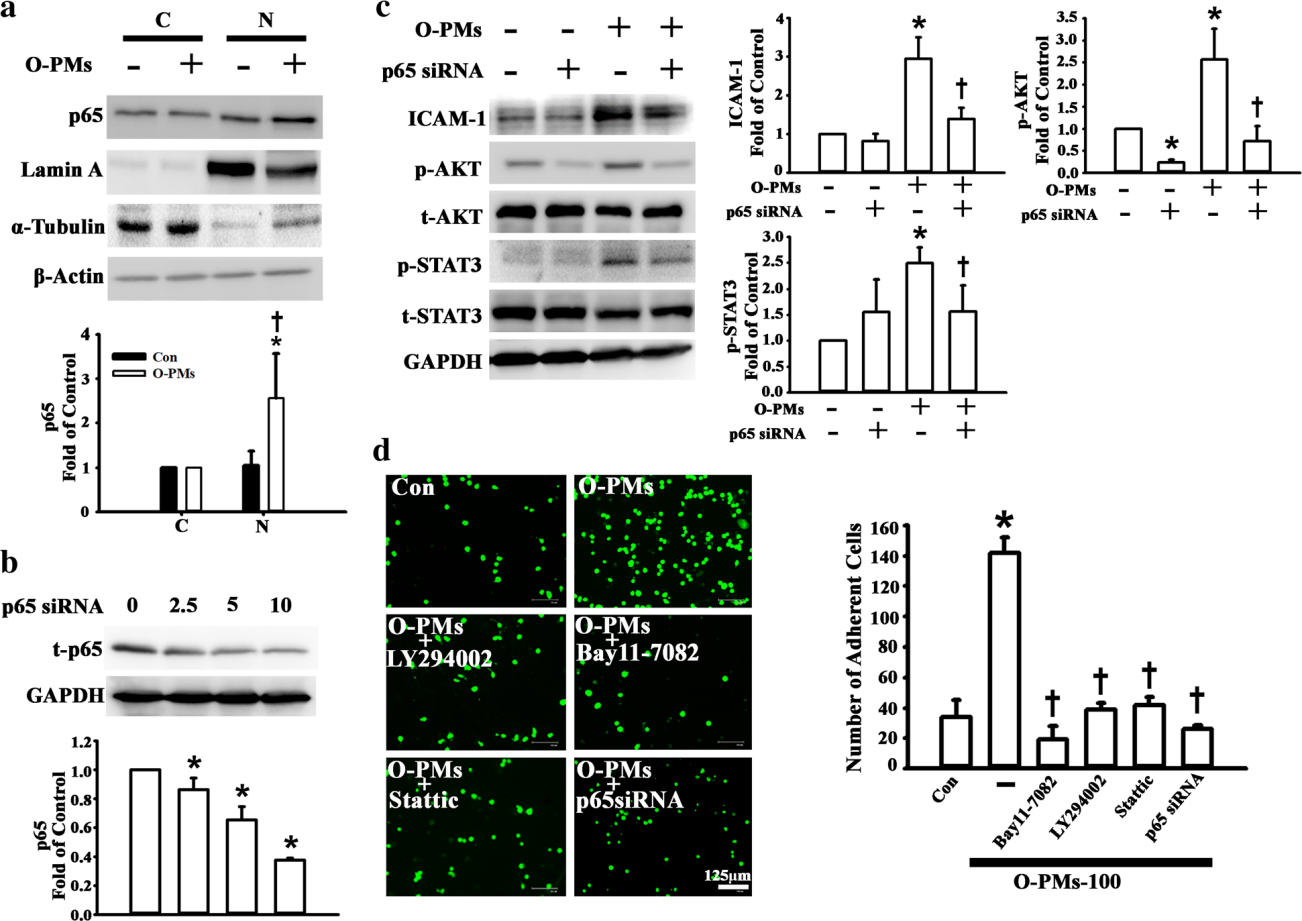
NF-κB activation was involved in O-PMs-induced ICAM-1 expression in A549 cells. **a** The effects of O-PMs treatment on translocation of NF-κB p65 in A549 cells. A549 cell were treated with or without 100 μg/ml O-PMs for 24 h. The distribution of p65 in the cytosolic (C) and nuclear (N) portion of A549 cells was determined by Western blot. *p < 0.05 vs. the cytosolic portion. †*p* < 0.05 vs. compared to the nuclear portion. **b** A549 cells were transfected with various concentrations of siRNA of p65 for 48 h. Levels of p65 were determined by Western blot. *p < 0.05 vs. 0 (untransfected cells). **c** After the transfection of p65 siRNA for 24 h, A549 cells were then stimulated with 100 μg/ml O-PMs for 24 h. The ICAM-1, p-AKT and p-STAT3 expression was determined by Western blot. *p < 0.05 vs. Con. †*p* < 0.05 vs. O-PMs. **d** Representative fluorescence photomicrographs and quantitative data showing the effects of AKT, NF-κB, and STAT3 on the adhesion of BCECF-AM-labeled U937 cells to O-PMs-treated A549 cells. Cells were pretreated for 1 h with BAY11–7082 (10 μM), LY294002 (10 μM), Stattic (10 μM) or p65 siRNA. Then they were treated with 100 μg/ml O-PMs for 24 h in the continued presence of the inhibitor. The cells without any treatment were used as the control (Con). BCECF-AM-labeled U937 cells were added to A549 cells and were incubated at 37 °C for 1 h. The adherent cells were photographed with a fluorescent microscope. Bar = 125 mu. *p < 0.05 vs. Con. †*p* < 0.05 vs. O-PMs

We next explored whether the NF-κB p65 translocation pathway is involved in O-PMs-induced ICAM-1 expression in A549 cells. To this aim, we used Western blot analysis to determine the expression levels of NF-κB p65 in the nuclear portion of A549 cells. The levels of p65 expression in nuclear portion were increased by O-PMs when compared with the control cells, but not in the cytosolic portion (Fig. 4a). To further confirm the involvement of NF-κB p65 in the O-PMs-induced ICAM-1 expression, we used p65-specific siRNA transfection to knockdown the p65 expression in A549 cells. The expression level of NF-κB p65 was downregulated by siRNA transfection for 48 h (Fig. 4b). Moreover, the levels of O-PMs-induced ICAM-1 expression as well as the AKT and STAT3 phosphorylation were attenuated in the p65 siRNA-treated A549 cells (Fig. 4c). A549 cells pretreated with 100 μg/ml of O-PMs for 24 h showed substantially higher monocyte adhesion than control cells (Con). The adherence of U937 cells to O-PMs-treated A549 cells was also markedly inhibited by the introduction of LY294002, BAY11–7082, Stattic or p65 siRNA (Fig. 4d).

### IL-6 was involved in O-PMs-induced ICAM-1 expression

**Fig. 5:**
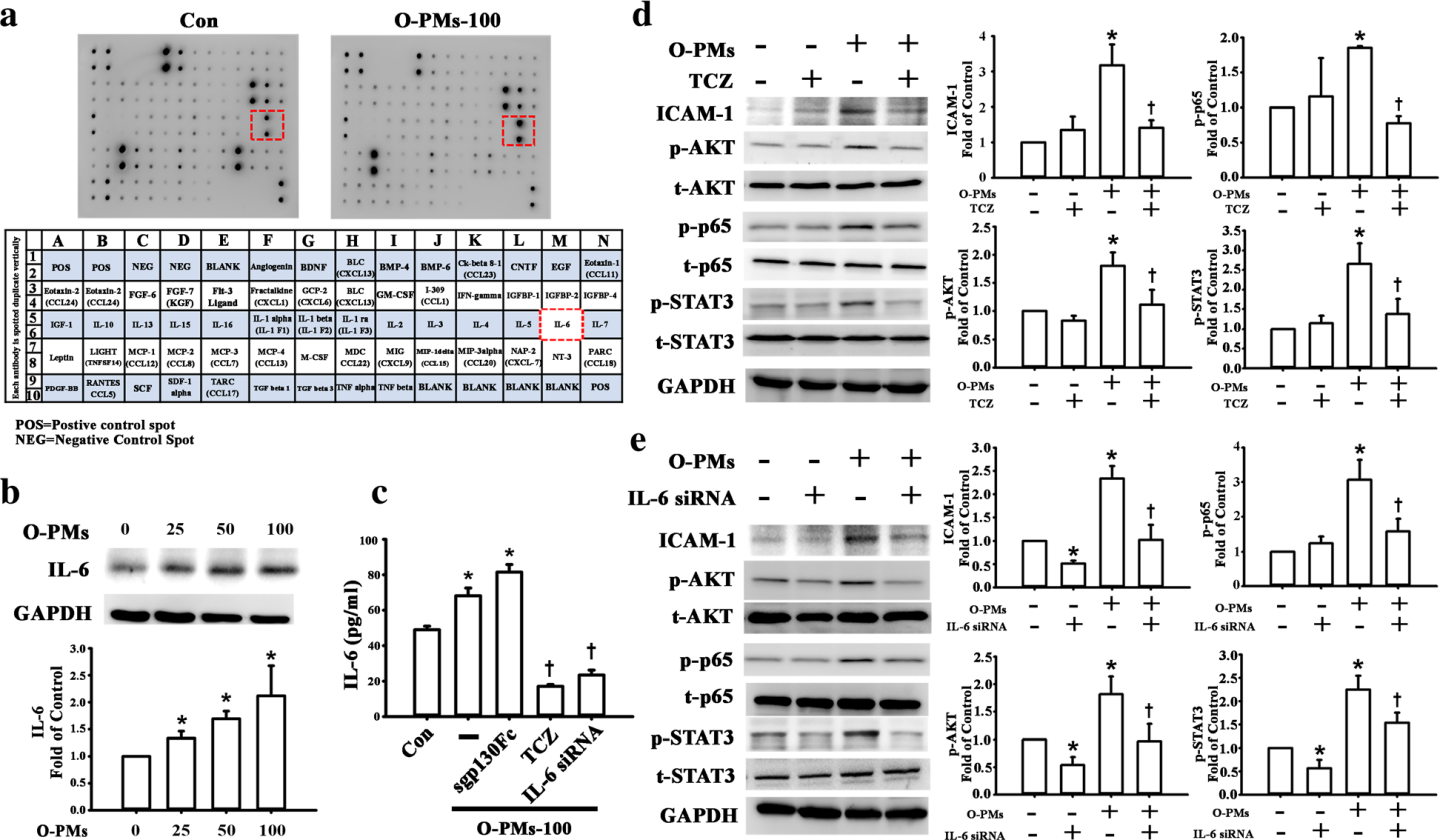
IL-6 was involved in O-PMs-induced ICAM-1 expression. **a** Representative photographs of the cytokine antibody array hybridized with conditioned media from A549 cells with or without 100 μg/ml O-PMs treatment for 24 h. **b** A549 cells were exposed to 0, 25, 50, or 100 μg/ml O-PMs for 24 H*. IL*-6 protein in cell lysates was measured by Western blot as described in the Methods section. GAPDH was used as the loading control. **c** A549 cells were pretreated with 20 μg/ml TCZ or with 5 μg/ml of sgp130Fc or with IL-6 siRNA, respectively, and then with treated with 100 μg/ml O-PMs for 24 H*. IL*-6 concentration in conditioned media was determined by ELISA. *p < 0.05 vs. Con. †*p* < 0.05 vs. O-PMs. **d**-**e** The effects of TCZ (**d**) and IL-6 (**e**) on the expression of ICAM-1 expression and the phosphorylation of AKT, p65 and STAT3. *p < 0.05 vs. the untreated cells. †*p* < 0.05 vs. O-PMs

To investigate the mechanisms by which O-PMs enhanced ICAM-1 expression in A549 cells, the human cytokine antibody array was used to screen the molecules secreted from O-PMs-treated A549 cells. Antibody array blot images and the corresponding lists of the tested cytokines are shown in the Fig. 5a. IL-6 was the most significantly changed cytokine between O-PMs-treated and O-PMs-untreated A549 cells (see Additional file 1: Table S1 online). IL-6 is known to play a crucial role in acute inflammation and to be induced in response to environmental insults to the lung injury [27, 28]. Thus, we further measured IL-6 levels in the cell lysates and in the conditioned media by Western blot and ELISA, respectively. As shown in Fig. 5b and c, 25–100 μg/ml of O-PMs exerts an increase in IL-6 expression and secretion.

Next, we sought to identify whether the differential IL-6 signaling pathways that are involved in the IL-6 production from O-PMs-treated A549 cells. sgp130Fc specifically blocks IL-6 trans-signaling without affecting IL-6 classic signaling [29], but it did not affect ICAM-1 expression in cells according to Western blot (see Additional file 1: Fig. S1 online). The IL-6 receptor inhibitor tocilizumab (TCZ) significantly reduced ICAM-1 secretion and expression in O-PMs-treated A549 cells (Fig. 5c and d). Furthermore, TCZ treatment also reduced the phosphorylation of AKT, p65, and STAT3 (Fig. 5d). IL-6 is also known to have the ability to induce ICAM-1 [30]. To explore whether IL-6 was involved in the O-PMs-induced ICAM-1 expression, we used IL-6-specific siRNA transfection to knockdown the IL-6 expression in A549 cells (Fig. 5c). Moreover, the level of O-PM-induced ICAM-1 expression and the phosphorylation of AKT, p65, and STAT3 were also attenuated in the IL-6-siRNA-treated A549 cells (Fig. 5e).

### PMs-induced ICAM-1 and IL-6 expression in lung tissues of WT mice

**Fig. 6:**
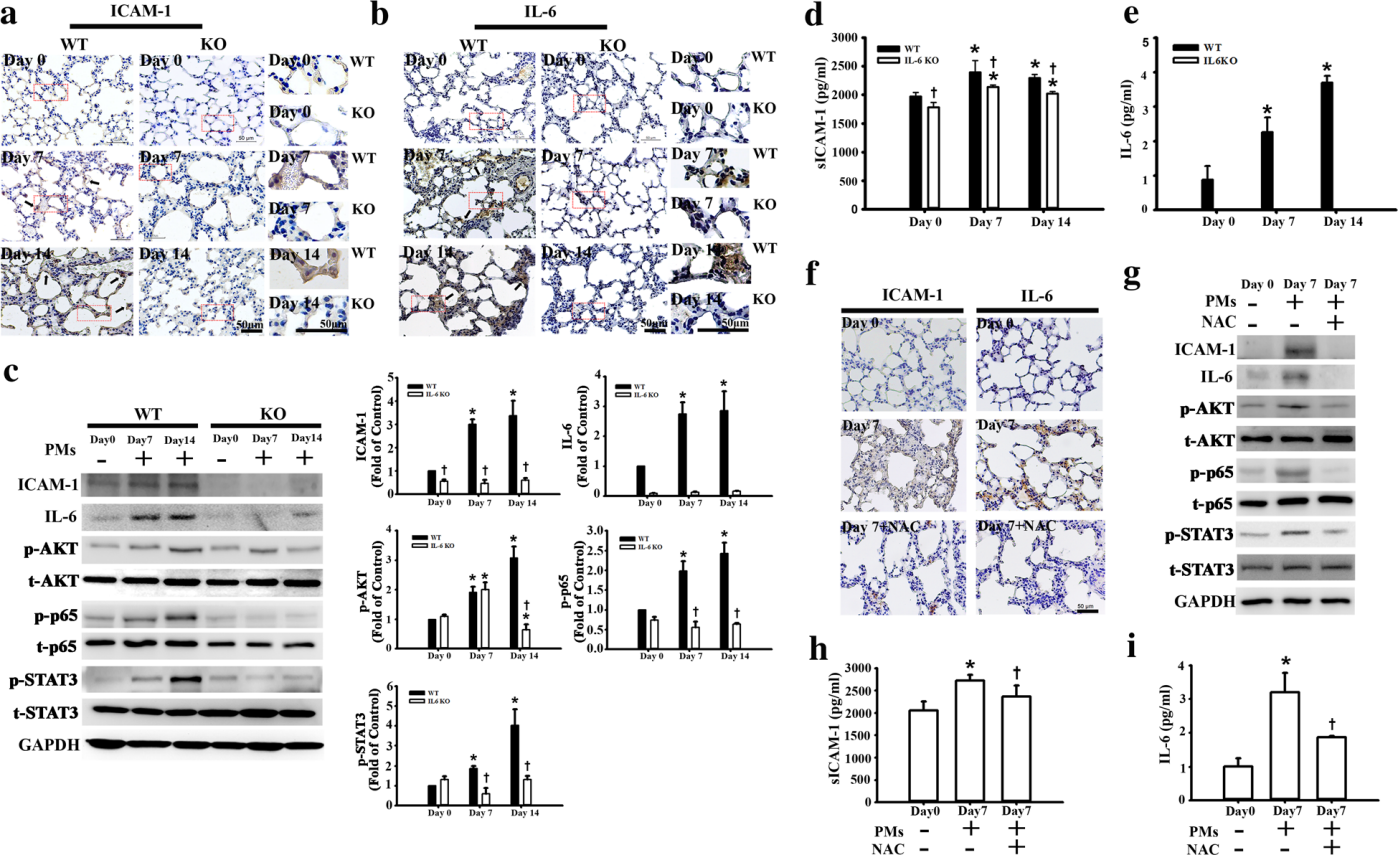
PMs-induced ICAM-1 and IL-6 expression in lung tissues of WT mice. WT and IL-6 KO mice were intratracheal instillation with 200–350 mg/mouse PMs for 7 and 14 days. Mice were euthanized, and plasma and lung tissues were collected at the determined time. **a**-**c** The levels of ICAM-1and IL-6 expression in lung tissues of WT and IL-6 KO mice were examined by immunohistochemical staining (**a**-**b**) and Western blot (**c**). The reaction product is indicated by arrows. The boxed regions were enlarged and shown in the right panel. Bar = 50 mu. **d**-**e** The levels of sICAM-1 and IL-6 in plasma were measured by ELISA. Data are expressed as mean ± SEM. (*n* = 6 for each group). *p < 0.05 vs. WT or IL-6 KO at Day 0, respectively. †*p* < 0.05 vs. WT at Day 7 or Day 14. WT mice (n = 6) were treated NAC (3–5 mg/mouse, 150 mg/kg body weight) before PM intratracheal instillation (200–350 mg/mouse). **f** The levels of ICAM-1and IL-6 expression in lung tissues were examined by immunohistochemical staining. **g** The expression of ICAM-1 and IL-6 as well as the phosphorylation of AKT, p65 and STAT3 in lung tissues were examined by Western blot. **h**-**i** The levels of sICAM-1 and IL-6 in plasma were measured by ELISA. *p < 0.05 vs WT at Day 0. †*p* < 0.05 vs Day 7

To detect the effects of PMs on ICAM-1 expression under inflammation in vivo, WT and IL-6 KO mice were untreated or injected intratracheally with PMs (200–350 μg/mouse) for 7 days and 14 days. The lung tissues of PMs-treated mice were examined by immunohistochemical staining and Western blot. As shown in Fig. 6a and b, PMs significantly induced ICAM-1 and IL-6 expression in lung tissues at Day 7 and Day 14. However, in IL-6 KO mice, the expression of ICAM-1 and IL-6 was not obvious according to immunohistochemical staining. PMs significantly induced the expression of ICAM-1 and IL-6 as well as the phosphorylation of AKT, p65, and STAT3 in lung tissues by Western blot (Fig. 6c). To ascertain whether lung injury is associated with intratracheal instillation of PMs, we examined plasma sICAM-1 and IL-6 levels in WT and IL-6 KO mice. Plasma concentrations of sICAM-1 and IL-6 were higher in the WT mice with or without PMs treatment compared with those in the IL-6 KO mice at Day 7 and Day 14 (Fig. 6d and e). Next, we investigated the role of ROS in PMs-induced pulmonary inflammation, pre-administration of NAC (3–5 mg/mouse) before intratracheal instillation of PMs (200–350 μg/mouse, 7 days) significantly attenuated PMs-induced ICAM-1 and IL-6 expression in lung tissues by immunohistochemical staining (Fig. 6f). Furthermore, NAC attenuated PMs-induced the expression of ICAM-1 and IL-6 as well as the phosphorylation of AKT, p65, and STAT3 by Western blot (Fig. 6g). Moreover, NAC attenuated the release of PMs-induced sICAM-1 and IL-6 in plasma (Fig. 6h and i).

### The higher levels of white blood cells (WBCs) count, C-reactive protein (CRP), sICAM-1, and IL-6 in COPD patients

**Fig. 7:**
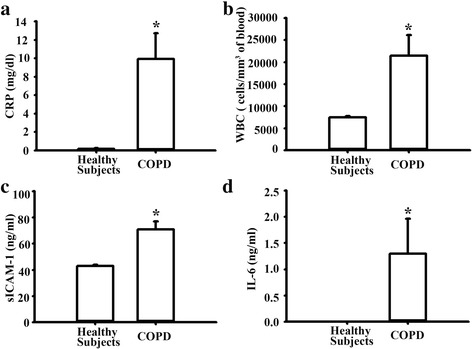
Plasma levels of WBC, CRP, sICAM-1, and IL-6 in COPD patients and healthy subjects. **a**-**b** Plasma levels of white blood cell (WBC) and C-reactive protein (CRP) levels. **c**-**d** The plasma level of sICAM-1 and IL-6 were assayed by ELISA. Healthy subjects (*n* = 8) and COPD patients with smoking history (n = 8) were enrolled. *p < 0.05 vs. healthy subjects

COPD is characterized by lung inflammation and ultimately results in the development of airflow obstruction [31]. COPD patients with a smoking history were closely associated with the inhaled component of PM2*.*5 from smoking cigarettes [32]. There were seven males and one female in the COPD group. The median age was 77 years (range 49–88). There were six males and two females in healthy subjects with normal pulmonary function. The median age was 68 years (range 51–82). Their smoking status, plasma sICAM-1 and IL-6 levels were listed in Table 1. The plasma levels of WBC and CRP also showed a highly statistically significant increase in COPD patients compared to the healthy subjects (Fig. 7a and b). Moreover, COPD patients had higher plasma levels of sICAM-1 and IL-6 compared to healthy subjects (Fig. 7c and d). Consistent with the results from the animal model, the plasma levels of sICAM-1 and IL-6 were significantly high in plasma from COPD patients.

**Table 1:** Smoking status, plasma sICAM-1 and IL-6 levels in COPD patients and in healthy subjects

Group I	Age	Gender	Smoking status	sICAM-1 (ng/ml)	IL6 (ng/ml)
C1	82	M	Ex-smoker 20 pack-years	67.73	5.29
C2	87	M	Ex-smoker 35 pack-years	40.19	0.29
C3	73	M	Ex-smoker 20 pack-years	62.95	1.11
C4	49	M	Active smoker30 pack-years	78.08	0.11
C5	81	M	Ex-smoker 15 pack-years	59.40	0.044
C6	88	F	Ex-smoker 12 pack-years	84.41	3.34
C7	70	M	Ex-smoker 38 pack-years	95.68	0.435
C8	74	M	Active smoker 33 pack-years	79.19	0.012
Group II					
H1	67	M	Never-smoker	43.81	0
H2	61	M	Never-smoker	46.81	0
H3	68	M	Never-smoker	40.84	0
H4	69	M	Never-smoker	43.81	0
H5	82	M	Never-smoker	42.84	0
H6	76	M	Never-smoker	41.74	0
H7	65	F	Never-smoker	45.33	0
H8	73	F	Never-smoker	39.11	0

## Discussion

This study demonstrates that PMs treatment increases both ICAM-1 expression in A549 cells and monocyte adhesion to these cells, along with the effects involved in ROS production, AKT/STAT3 phosphorylation, and NF-κB activation. The effect was also mediated through the upregulation of IL-6 expression. Moreover, TCZ and siIL-6 attenuated the O-PMs-induced ICAM-1 expression and phosphorylation of AKT/STAT3/NF-κB. Furthermore, we demonstrated that ICAM-1 and IL-6 expression were markedly increased in lung tissues and in the plasma of PMs-treated WT mice. PMs had little effect on ICAM-1 expression in IL-6 KO mice. In addition, higher plasma levels of ICAM-1 and IL-6 were also found in COPD patients. Taken together, these results suggest that PMs-induced ROS may contribute to ICAM-1 expression by activating the IL-6/AKT/STAT3/NF-κB signaling pathways in the epithelial cells of the lungs.

Epidemiological studies have demonstrated that PM_2.5_ is a serious environmental contaminant and is responsible for multiple human diseases, including pulmonary diseases and cardiovascular diseases [33, 34]. PM_2.5_ exposure increases intracellular ROS generation in primary cultured human umbilical vain endothelial cells, human microvascular endothelial cells, and lung epithelial cells [35, 36]. Consistent with these reports, both O-PMs and W-PMs significantly increased ROS production in the present study. Importantly, the cytotoxicity, ROS generation and monocyte adherence to A549 cells were more severely affected by O-PMs than by W-PMs treatment. In addition, O-PMs significantly increased ICAM-1 expression in A549 cells, but not W-PMs. Similarly, a previous report showed that organic extracts from motorcycle exhaust particles containing PAH and induced ICAM-1 expression in endothelial cells [37]. The chemical composition of SRM1649b PMs is involved in biological and toxicological effects, including both the water-soluble and the organic solvent-soluble fractions [38, 39]. After inhalation, the water-soluble fraction mainly infiltrates the pulmonary surface, which is involved in hydroxyl radical production in abiotic conditions. The organic solvent-soluble fraction could initiate a cascade of intracellular signaling pathways [40], such as inflammatory cytokine production (IL-6, IL-8) and aryl hydrocarbon receptor (AhR)-dependent signaling (CYP1A1) [41]. The organic solvent-soluble fraction was found to cause a higher ROS response, which was mainly associated with cytotoxic and inflammatory effects [42]. The certificate of analysis for SRM1649b used in this study has been reported previously. The water-soluble contents are primarily composed of low-molecular-weight ions (Ni, Cu, Cr, Mn, V, Fe, Al, Ca, Na, K, Mg, Pb, Cl^−^, F^−^, SO_4_^2−^, and NO3^−^) [43]. The organic extractable fraction contains polycyclic aromatic hydrocarbons, steranes, and hopanes [44]. We did not examine the difference in the components between O-PMs and W-PMs. However, it would be necessary to identify whether the components in O-PMs induce ICAM expression in pulmonary epithelial cells in the future. Previous reports demonstrated that ROS act as intracellular messengers to induce the expression of cell adhesion molecules in epithelial cells [45, 46]. NAC acts as a source of cysteine and stimulates the production of glutathione, which protects against oxidative damage. Indeed, administration of NAC often causes a time-dependent increase in the cell content of glutathione, the most abundant thiol antioxidant [47]. The present study is the first to report that pretreatment with NAC significantly reduced O-PMs-induced ICAM-1 expression and monocyte adhesion to epithelial cells, indicating that O-PMs-induced ROS were involved in stimulating the expression of adhesion molecules.

The MAPK or AKT pathways played an important role in the proinflammatory mediator expression and the inflammatory cell recruitment, which led to the initiation and progression of lung inflammation [48]. In the present study, O-PMs caused a significant increase in AKT phosphorylation and ICAM-1 expression. This increase of ICAM-1 expression in the cell lysates was markedly inhibited in the presence of an AKT inhibitor. Thus, one of the mechanisms by which O-PMs increases ICAM-1 expression involves the induction of AKT activation in pulmonary epithelial cells. Consistent with our results, PM_2.5_ has been shown to induce ICAM-1 expression in human endothelial cells by activation of AKT [49]. Interestingly, our study showed that O-PMs did not cause a marked activation of three MAPK subtypes (ERK, JNK, and p38) in A549 cells. However, another study found that PM activates JNK to induce alveolar epithelial cell death [50]. PM_2.5_ decreases cell viability and increases apoptosis by activating the MAPKs pathway in H9c2 cells [51]. PM_2.5_ induced amphiregulin expression, a ligand of the epithelial growth factor receptor, by ERK but not by the p38 pathway in human bronchial epithelial cells [52]. The differences between these results in terms of the pathways involved may be related to differences in cell types, cytokines, or inducers.

NF-κB is a transcription factor that regulates a wide variety of biological reaction in response to oxidative stress in different types of cells and organs [53]. Multiple signaling pathways including the PKC, PKA, MAPK, and AKT pathways act directly to phosphorylate NF-κB subunits to affect the NF-κB translocation and regulate the transactivation of NF-κB-dependent genes [54]. The induction of ICAM-1 gene transcription has previously been shown to be dependent on NF-κB activation and its binding to ICAM-1 promoter [55]. Our data showed that O-PMs upregulated NF-κB p65 phosphorylation and increased the p65 translocation into nucleus. Furthermore, we also demonstrated that cells treated with NF-κB inhibitor (BAY11–7082) or with siRNA p65 significantly reduced ICAM-1 expression after exposure to O-PMs. These observations are consistent with previous reports that NF-κB is responsible for the ICAM-1 and VCAM-1 expression in endothelial cells [49]. Furthermore, we demonstrated that the NF-κB p65 phosphorylation was significantly attenuated after pretreatment with NAC. In addition, NF-κB inhibitor reduced the adhesion of monocytes to A549 cells after exposure to O-PMs. The results indicated that ROS generation is associated with the activation of the NF-κB pathway, which contributes to ICAM-1 expression and cell adhesion after O-PMs exposure. Potential sources of ROS production following exposure to PM include the mitochondria, cell membranes, phagosome of inflammatory cell with NADPH oxidase activation, and the endoplasmic reticulum [56]. In addition, the organic extracts of dust remarkably suppressed antioxidant enzymes (superoxide dismutase and catalase) in human epithelial cells [57]. The source of O-PM_2.5_-induced ROS production and the O-PM_2.5_-affected antioxidant enzyme expression in our study have yet to be elucidated and warrant further investigation.

Epithelial lung cells exposed to PM_2.5_ led to significant increases in protein secretion or gene expression of inflammatory cytokines, including IL-6, IL-1β, and TNF-α [58]. Airborne particles markedly evoked IL-6 production in the transformed bronchial epithelial cells [59]. The PM in residual oil fly ash increased IL-6 secretion from lung macrophages [60]. Consistent with a previous study, our data showed that IL-6 was upregulated after O-PMs exposure. In addition, we also demonstrated that TCZ significantly decreased O-PMs-induced IL-6 secretion, whereas sgp130Fc had no effects on the secretion. This observation is consistent with a previous report that IL-6 trans-signaling is proinflammatory, whereas classic IL-6 signaling is needed for regenerative or anti-inflammatory activities of the cytokine [29]. Moreover, we also found that TCZ or siRNA IL-6 significantly decreased O-PMs-induced ICAM-1 expression as well as AKT, p65, and STAT3 phosphorylation. Further validation of these in vitro findings was carried out in vivo using a well-established IL-6 KO mouse model. The immunohistochemical staining of mouse lung tissues revealed a notable increase in ICAM-1 expression specific to the epithelial layer of the PMs-treated WT mice, whereas IL-6 KO mice showed little effect on PMs-induced ICAM-1 expression.

Elevated levels of IL-6 are a primary stimulant of sICAM-1, which increases in response to inflammatory stimuli and injury in endothelial cells [61]. The sICAM-1 is a predictor of cardiovascular diseases in healthy individuals. It acts as an activator for lung macrophages, and it enhances lung injury [62, 63]. Furthermore, the present study demonstrated that the plasma levels of sICAM-1 were lower in IL-6 KO mice than in WT mice, suggesting that IL-6 is an essential inducer of ICAM-1 expression during inflammation. We also showed that the plasma levels of sICAM-1 and IL-6 of WT mice were higher than those in IL-6 KO mice in the PMs-exposed groups at Day 7 and Day 14. NAC has been known as an antioxidant and anti-inflammatory agent [64]. In particular, NAC prevented lung inflammation in mice after short-term inhalation of concentrated ambient particles [65]. NAC administration reduced both the systemic oxidative stress and the recruited immune cells to the lung after coarse PMs inhalation [66]. The present study demonstrated that PMs-induced adverse effects was significantly attenuated after pre-administration of NAC in vitro and in vivo*.*

The elevated levels of ICAM-1 and IL-6 expression were also demonstrated in plasma in COPD patients with a smoking history. It is difficult to collect blood samples from ambient PMs-induced COPD patients. The previous studies estimated that smoking a single cigarette was equivalent to breathing a daily ambient concentration of PM2.5 of 667 μg/m^3^, assuming an average breathing rate of 18 m^3^/day and an inhaled dose of 12,000 μg PM_2.5_ mass per cigarette [67, 68]. Cigarette smoking often results in the development of COPD (Schroeder et al., 2013). So patients who had a long-term smoking history (≥10 pack-years) and were diagnosed with COPD instead of PMs-inhaled humans were enrolled into the current study. Our findings provide the evidence that the both sICAM-1 and IL-6 levels were remarkably high in COPD patients than those in healthy subjects and these results were similar to those in PMs-treated mice. The previous study reported that ICAM-1 expression is upregulated in the epithelium of airways in both smokers and in patients with airflow obstruction [69]. Another study demonstrated that plasma IL-6 level was significantly higher in smoking-related COPD patients than in healthy subjects [70]. These data implied the elevated sICAM-1 and IL-6 levels in COPD patients with a smoking history were closely associated with the inhaled component of PM2.5 from smoking cigarettes.

The higher dose of PMs used in the present study may be explained by the short length of the incubation time. Consistent with our study, a number of reports used the similar amount of PMs. The doses of 100 μg/ml (in vitro) or 200–350 μg /mouse (10 mg/kg, in vivo) were commonly used in particulate matter toxicology studies [50, 71–74]. Future studies will use the low dose of PMs for longer incubation times, allowing to more accurately estimate the effects of inhaled urban air pollutant on human respiratory diseases.

## Conclusions

Extensive epidemiologic and experimental evidence has demonstrated that particulate air pollution directly causes lung inflammation. In conclusion, this study provides the first evidence that O-PMs-induced oxidative stress led to increased ICAM-1 expression both in vitro and in vivo. We also showed that the ICAM-1 expression occurred through activating IL-6/AKT/STAT3/p65 and promoted leukocyte adhesion to alveolar epithelial cells. Higher plasma levels of ICAM-1 and IL-6 were also found in COPD patients. Based on these findings, understanding the signaling pathways implicated in PMs-mediated inflammation provides new insights into the pathophysiology of airway diseases associated with airborne pollution.

## Additional file

Additional file 1: Table S1. Summary of results from the cytokines antibody array. IL-6 was the most significant changes in angiogenic factors between O-PMs-100 and CON and was selected for further analysis in the present study. **Figure S1.** The sgp130 Fc did not affect ICAM-1 expression in O-PM treated cells. A549 cells were pretreated with 5 μg/mL of GP130FC for 1 h and then treated with 100 μg/ml of O-PMs for 24 h. Cell lysates were blotted for ICAM-1 expression. (DOCX 108 kb)

## Abbreviations

BSA: Bovine serum albumin
CM: Conditional medium
COPD: Chronic obstructive pulmonary disease
CRP: C-Reactive protein
DAB: 3,3-diaminobenzidine tetrahydrochloride
DCFH-DA: 2′,7′-dicholorofluorescein-diacetate
DMEM: Dulbecoo’s Modified Eagle Medium
DMSO: Dimethyl sulfoxide
ELISA: Enzyme-linked immunosorbent assay
FBS: Fetal bovine serum
ICAM-1: Intercellular adhesion molecule-1
MAPK: Mitogen-activated protein kinases
MTT: 3-(4,5-dimethylthiazol-2-yl)-2,5 –diphenyltetrazolium bromide
NAC: N-acetyl cysteine
O-PM: Organic solvent-extractable fraction of SRM1649b
PAHs: Polycyclic aromatic hydrocarbons
PM: Particulate matter
ROS: Reactive oxygen species
SDS: Sodium dodecyl sulfate
TCZ: Tocilizumab
TNF-α: Tumor necrosis factor-α
WBC: White blood cells
W-PM: Water-soluble fraction
