# Supplementary material for: PM2.5-induced oxidative stress increases intercellular adhesion molecule-1 expression in lung epithelial cells through the IL-6/AKT/STAT3/NF-κB-dependent pathway
Source: Part Fibre Toxicol. 2018 Jan 12;15:4. doi: 10.1186/s12989-018-0240-x (PMC5767014; doi:10.1186/s12989-018-0240-x)
Supplement: Additional file 1: Table S1. — Summary of results from the cytokines antibody array. IL-6 was the most significant changes in angiogenic factors between O-PMs-100 and CON and was selected for further analysis in the present study. Figure S1. The sgp130 Fc did not affect ICAM-1 expression in O-PM treated cells. A549 cells were pretreated with 5 μg/mL of GP130FC for 1 h and then treated with 100 μg/ml of O-PMs for 24 h. Cell lysates were blotted for ICAM-1 expression. (DOCX 108 kb) [file 12989_2018_240_MOESM1_ESM.docx]

**Supplement**

**Title:** PM_2.5_-induced oxidative stress increases intercellular adhesion molecule expression in lung epithelial cells through the IL-6/AKT/STAT3/NF-κB-dependent pathway

**Authors:** Chen-Wei Liu, Tzu-Lin Lee, Yu-Chen Chen, Chan-Jung Liang, Shu-Huei Wang, June-Horng Lue, Jaw-Shiun Tsai, Shih-Wei Lee, Tzu-Yi Chuang, Yuh-Lien Chen


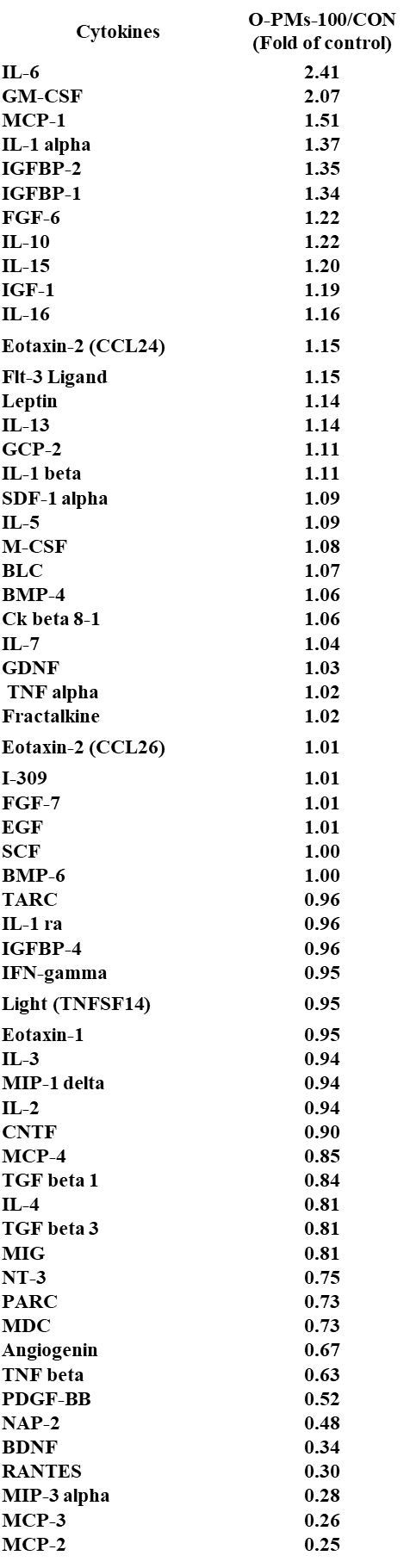


**Table S1: Summary of results from the cytokines antibody array.** IL-6 was the most significant changes in angiogenic factors between O-PMs100 and CON and was selected for further analysis in the present study.


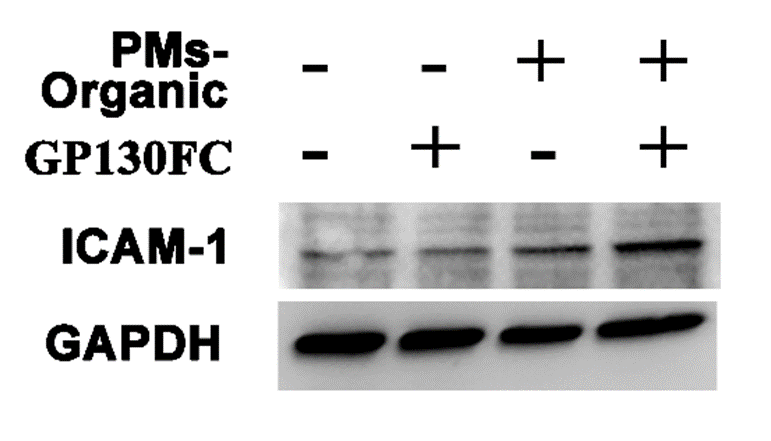


**Figure S1:** **The sgp130 Fc did not affect ICAM-1 expression in O-PM treated cells.** A549 cells were pretreated with 5μg/mL of GP130FC for 1 h and then treated with 100 μg/ml of O-PMs for 24h. Cell lysates were blotted for ICAM-1 expression.
